# New Sustainable Material for Metal Ions Removal: Adsorption Mechanism and Technological Innovations

**DOI:** 10.3390/polym18060712

**Published:** 2026-03-14

**Authors:** Luoana Florentina Pascu, Toma Galaon, Adriana Mariana Borș, Nicoleta Mirela Marin

**Affiliations:** 1 National Research and Development Institute for Industrial Ecology ECOIND, Street Podu Dambovitei No. 57-73, District 6, 060652 Bucharest, Romania; luoanapascu@yahoo.com (L.F.P.); tomagalaon@yahoo.com (T.G.); 2 Department of Analytical and Physical Chemistry, University of Bucharest, 4-12 Regina Elisabeta Bd., 030018 Bucharest, Romania; 3 National Institute for R&D for Optoelectronics-Subsidiary, Research Institute for Hydraulics and Pneumatics—INOE 2000-IHP, 040558 Bucharest, Romania; bors.ihp@fluidas.ro; 4 Department of Oxide Materials Science and Engineering, National University of Science and Technology Politehnica Bucharest, 1–7 Gh. Polizu, 060042 Bucharest, Romania

**Keywords:** metal ions, Alizarine Red S, maize stalk, adsorption, green chemistry, circular economy

## Abstract

In this study, a novel material was obtained by functionalizing shredded maize stalk (MS) with Alizarine Red S (ArS), a complexing agent that contains −OH and −C=O groups in its structure (MS-ArS). The obtained MS-ArS was employed in adsorption studies for Mn^2+^, Pb^2+^, Cu^2+^, Cr^3+^, Zn^2+^, and Fe^3+^ (M^n+^) removal from mixed aqueous matrices. Initially, complex formation between (M^n+^) and ArS in buffer solution at pH 4 and 10 was investigated using the UV-Vis spectrometric method. Continuous, the functionalization process of MS with ArS was tested at several pH values (2, 4, 6, 8, and 10) using a batch technique. It was observed that the best functionalization of MS with ArS was obtained at pH = 2. Subsequently, M^n+^ adsorption onto the MS-ArS mass was tested separately at pH 4 and 10. The study achieved that M^n+^ adsorption proved to be pH dependent. The results confirmed that at pH = 10, M^n+^ adsorption was increased, compared with pH = 4. MS-ArS has affinity for M^n+^ in the following order Fe^3+^ > Cu^2+^ > Zn^2+^ > Mn^2+^ > Pb^2+^ > Cr^3+^. Experimental data revealed remarkable desorption rates when 0.5 M HCl was used. After five adsorption/desorption cycles of M^n+^, the removal capability of MS-ArS was preserved. Overall, the potential of MS-ArS for effective M^n+^ removal/reuse makes it a sustainable polymer for wastewater treatment applications.

## 1. Introduction

Nowadays, wastewater treatment is a critical part of environmental remediation [1]. Human activities—particularly mining operations and the intensive use of fertilizers, herbicides, and pesticides—significantly contribute to heavy metal contamination. This issue is further intensified by the irrigation of agricultural land with untreated sewage and industrial waste [1,2,3]. Consequently, wastewater composition varies by source, typically containing pathogens besides suspended and dissolved organic and inorganic pollutants, all of which disrupt ecological stability [4,5]. The extensive contamination of water with heavy metals represents a critical challenge requiring special attention. Heavy metals, in contrast to organic pollutants, do not biodegrade over time [6,7,8]. Additionally, their potential for biological accumulation poses significant risks to aquatic organisms and, ultimately, human health, often manifesting as carcinogenic effects. Therefore, reduction in heavy metal pollution is essential for the protection of both environmental integrity and public health [5,9,10,11,12]. Involving effective strategies for the removal and recovery of heavy metals is vital for both the sustainable management of resources and the reduction in toxic environmental impacts [13,14,15,16,17,18,19,20,21,22,23]. Recent research has shifted toward employing eco-friendly technologies as sustainable alternatives to conventional ion exchange resins and classical treatment methods [24,25,26]. Developing these innovative methods requires a thorough understanding of adsorbent structures, including their physico-chemical properties and specific removal mechanisms. Furthermore, various analytical techniques are now employed to characterize the interaction between pollutants and adsorbent materials, ensuring more efficient remediation of contaminated effluents [13,27,28,29,30,31,32,33,34,35,36].

So, the aim of this study was to obtain a new functionalized cellulose material by which the shredded maize stalk was functionalized with ArS to achieve a novel complexing polymer for M^n+^ removal.

To investigate the M^n+^ adsorption, a series of experiments was conducted using MS-ArS. Experimental parameters like pH influence, contact time, initial metal ions concentration, and desorption/reutilization were studied. Also, the equilibrium data were fitted taking into consideration kinetics and isotherm models in order to quantify M^n+^ adsorption onto MS-ArS mass. The FTIR technique was also employed for the characterization of the solid phase of MS, MS-ArS, and MS-ArS-M^n+^.

## 2. Materials and Methods

### 2.1. Reagent

ArS (CAS 130–22-3, Carl Roth, Karlsruhe, Germany) was used as a reagent for complexing shredded maize stalk. Mono-element solutions of 1000 mg/L Mn(NO_3_)_2_, Pb(NO_3_)_2_, Cu(NO_3_)_2_, Cr(NO_3_)_3_, Zn(NO_3_)_2_, and Fe(NO_3_)_3_ from Merck (Darmstadt, Germany), were used for preparing calibration curves and for obtaining solutions used in adsorption experiments. NaCl solid (for analysis) and 37% HCl were purchased from Merck and were used in the purification of maize stalk and regeneration experiments. KBr solid was used for FTIR analysis (Merck).

### 2.2. Equipment

For UV-VIS spectrometric analysis, an Agilent Cary 60 UV–Vis spectrophotometer (Penang, Malaysia) was employed. For the metal ions analysis, a Perkin Elmer PinAcle 900T atomic absorption spectrometer (Perkin Elmer, Norwalk, CT, USA, in flame mod using air-C_2_H_2_ method) was utilized. The HI 255 pH-meter (Hanna Instruments, Nijverheidslaan, Belgium) was used for pH measurement of the supernatant and buffer solutions. For the preparation of the complexing material and for metal ions adsorption, a GFL Type 3017 LAUDA horizontal mechanical shaker (Burgwedel, Germany) was used.

### 2.3. Experimental Methodology Used in Adsorption Study

In this paper, all experimental data were conducted in duplicate, and only the average values were employed to represent the experimental data given in Results and Discussion presented in Section 3. Furthermore, synthetic samples were developed to closely simulate environmental conditions, allowing us to thoroughly assess the performance of the complexing polymer produced.

### 2.4. Procedures for Testing the Complex Formation Between ArS and M^n+^ in Solution at Different pH Buffer

UV-Vis spectrum of ArS was recorded on an ArS solution prepared by combining 0.75 mL ArS (500 mg/L) with 0.75 mL buffer solution at pH 4 in a 10 mL volumetric flask. After mixing, ultrapure water was added to bring the total volume to the mark. For the recorded UV-Vis ArS-M^n+^ spectrum, the mixed solution of ArS-M^n+^ was obtained by adding 0.5 mL of M^n+^ (100 mg/L), which was combined with 0.75 mL of ArS (500 mg/L) and 0.75 mL of acetate buffer at pH 4 in a 10 mL volumetric flask, which was then filled to the mark with ultrapure water. Each solution after preparation was placed in a cuvette of a UV-Vis spectrometer, and the spectra were recorded after 10 min of reaction in the 200–800 nm range. The same experimental procedure was followed to study complex formation at pH 10.

### 2.5. Procedure for Functionalization of Shredded Maize Stalk with ArS at Different pH Values

Over 0.05 g MS, 1.5 mL ArS 500 mg/L, and 1.5 mL buffer solutions, together with 7 mL of ultrapure water, were added to Erlenmeyer flasks.

For functionalization of shredded maize stalk with ArS, the buffer solutions tested were: pH = 2.0 (phosphate buffer); pH = 4.0 (acetate buffer); pH = 6.0 (acetate buffer); pH = 8.0 (phosphate buffer), and pH = 10 (carbonate buffer).

The obtained mixtures were stirred at 175 rpm, 60 min at T = 25 ± 2 °C. At the end of the stirring, the mixtures were filtered, and the amount of ArS that was not retained on the cellulose mass was spectrometrically determined. Shredded maize stalk was prepared for functionalization as presented in a previous study [37]. The UV-Vis method used in this study has been tested in our laboratory. Solutions for detecting UV-Vis analytical linearity were obtained by diluting the 500 mg ArS solution to obtain a concentration range from 12, 18, 24, 36, to 48 mg/L. For this purpose, each standard solution was analyzed, and the linear regression line y = 23.453x + 0.0223 was obtained. The detection and determination limits of the UV-Vis method were 0.02 mg/L and 0.05 mg/L.

The adsorption capacity (*Q_e_*) as well as the percentages of ArS retained on the MS (R(%)), using Equations (1) and (2), were determined.

(1) $$Q_{e\;}=\frac{\left(C_{i}-C_{e}\right)\;V}{m}$$

(2) $$R\;\left(\%\right)=\frac{C_{i}-C_{e}}{C_{i}}\times 100$$

where *C_i_* represents the initial concentration of ArS and *C_e_* (mg/L) is the concentration at equilibrium, *m* (g) is the mass of dry MS, and *V* (L) is the volume of ArS used in the adsorption experiment.

### 2.6. Procedures Used to Evaluate the Influence of pH on M^n+^ Adsorption onto MS-ArS

Samples of 0.05 g MS-ArS were weighed and transferred into 100 mL Erlenmeyer flasks. Subsequently, 0.01 L buffer solution (pH = 4 and 10) containing 3.5 mg/L M^n+^ was added to the MS-ArS samples and stirred at 175 rpm (T = 25 ± 2 °C) for 60 min. At the end of the stirring time, the samples were filtered, and the liquid phase was kept for M^n+^ determination. The concentration of heavy metal ions from filtrate solution has been performed in the 0.1–0.5 mg/L range using a calibration curve for all six M^n+^ tested. The buffer solutions used were the acetate buffer for pH = 4 and the carbonate buffer for pH = 10, respectively. The M^n+^ studied in this article are the most harmful pollutants and are of interest due to their toxicity to the aquatic environment. Also, the physical and chemical properties of M^n+^ studied, which can be taken into consideration in adsorption studies, are presented in Table 1 [37].

### 2.7. Procedure for Adsorption of Mn^2+^, Pb^2+^, Cu^2+^, Cr^3+^, Zn^2+^ and Fe^3+^ onto MS-ArS at pH = 10 in Function of Contact Time

Samples of 0.05 g MS-ArS were weighed on an analytical balance and transferred into 100 mL Erlenmeyer flasks. Then, 0.01 L solution of 3.5 mg/L M^n+^ was added to MS-ArS samples, and the pH was adjusted by adding 0.75 mL carbonate buffer solution of pH = 10. The mixtures obtained were stirred at different intervals of times that was ranging from 5, 15, 30, 45, and 60 min, at 175 rpm (T = 25 ± 2 °C). At the end of each stirring time, samples were filtered, and all filtrates were analyzed using the AAS method to detect metal ion concentration that was not retained on the complexing material. The quantity of M^n+^ adsorbed at time (t), *Q_t_* (mg/g), on the MS-ArS mass was determined using Equation (3).

(3) $$Q_{t\;}=\frac{\left(C_{i}-C_{e}\right)\;V}{m}$$

where *C_t_* (mg/L) represents the concentration of metal ions in the solution at time *t*.

### 2.8. Procedure for Adsorption of Mn^2+^, Pb^2+^, Cu^2+^, Cr^3+^, Zn^2+^ and Fe^3+^ Using MS-ArS at pH = 10

For adsorption of Mn^2+^, Pb^2+^, Cu^2+^, Cr^3+^, Zn^2+^, Fe^3+^ onto MS-ArS, ≈ 0.05 g of MS-ArS (14.2 mg ArS/g) were stirred with 0.01 L solution, in which the concentration of Mn^2+^, Pb^2+^, Cu^2+^, Cr^3+^, Zn^2+^, Fe^3+^ was varied from 0.5; 1.5; 2.5; 3.5; to 5 mg/L and the pH was adjusted by adding 0.75 mL carbonate buffer solution of pH = 10. The mixtures were stirred for 45 min at 175 rpm (T = 25 ± 2 °C). After stirring, the mixtures were filtered, and the metal concentration was determined by atomic absorption spectrometry (AAS).

### 2.9. Procedures for Metal Ions Desorption and MS-ArS Regeneration

Samples of 0.05 g MS-ArS loaded with 0.65 mg Mn^2+^, 0.54 mg/g Pb^2+^, 0.83 mg/g Cu^2+^, 0.41 mg/g Cr^3+^, 0.75 mg/g Zn^2+^ and 0.87 mg/g Fe^3+^ were stirred for 30 min at 175 rpm (T = 25 ± 2 °C) with 0.01 L (0.5 M HCl, NaCl and hot water (H_2_O) temperature of water during experiment was kept at 50 ± 2 °C). After the desorption study, the concentration of M^n+^ was determined using the AAS method, and the desorption rate was calculated. Also, from the supernatant solution, the ArS concentration was detected by the UV-Vis method.

### 2.10. Procedures for the Reutilization of Complexing Material

Complexing material: 0.05 g of MS-ArS (loaded with 14.2 mg ArS/g) was stirred with 10 mL of 5 mg/L M^n+^ for 60 min at 175 rpm (T = 25 ± 2 °C). At the end of the stirring time, the mixture obtained was filtered, and the solid phases loaded with M^n+^ were kept for regeneration studies. Solid phases subsequently obtained were stirred with 0.5 M HCl (0.01 L) at 175 rpm (T = 25 ± 2 °C). At the end of the experiment, samples were filtered, and the concentration of M^n+^ from the filtrate solution was detected using the AAS method. The reuse procedure was tested up to 5 times (C1–C5) of adsorption/desorption studies on the same matrix of complexing material.

### 2.11. Characterization of Solid Samples

Characterization of MS, Ms-ArS, and MS-ArS-M^n+^ samples was carried out using a VERTEX 70 FTIR spectrometer (Bruker, Ettlingen, Germany) equipped with an ATR diamond accessory operating at a frequency of 4 cm^−1^ with 128 scans. The FTIR spectra were obtained in quadruplicate to ensure reliability and reproducibility. An atmospheric background measurement was performed prior to sample acquisition to correct for environmental interference. The highest-quality spectrum was automatically selected based on its ranking by OPUS 7.0 software and used for subsequent analysis to ensure optimal data accuracy and interpretation.

## 3. Results and Discussion

The ability of metal ions to form complexes with different ligands and the ability of the ligands to be selective under established experimental conditions, as well as the characterization of the resulting complex structure, is a continuous research interest topic. It is also known that metal ions, regardless of their position in the periodic table, can form complexes. This is based on the observation that a coordinate bond is formed between chemical species that accept (metal ion) and, respectively, donate electrons to the ligand (organic compound).

Starting from the premise that complex processes in solution exhibit similarities to those occurring in solid materials, this study was initiated by first examining the formation of complexes in solution as a function of the pH medium. This initial investigation provided a robust foundation for the subsequent analysis, which focused on the adsorption characteristics of six metal ions in the aqueous medium following the functionalization of MS with ArS. This comprehensive approach enhances our understanding of complexation studies and their applications in material science [38].

### 3.1. Studies on the Complex Formation (ArS-M^n+^) in Buffer Solutions at pH 4 and 10

**Figure 1:**
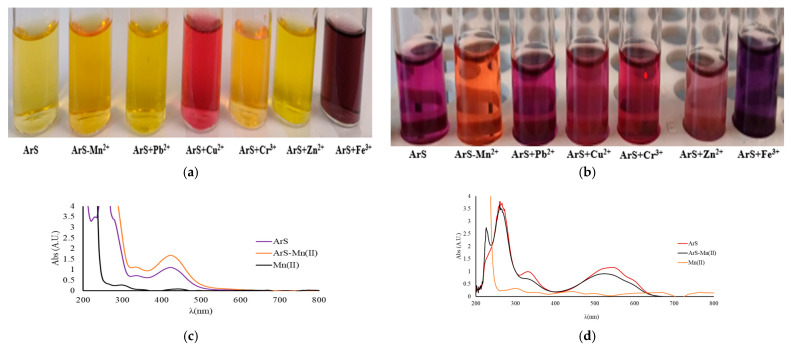

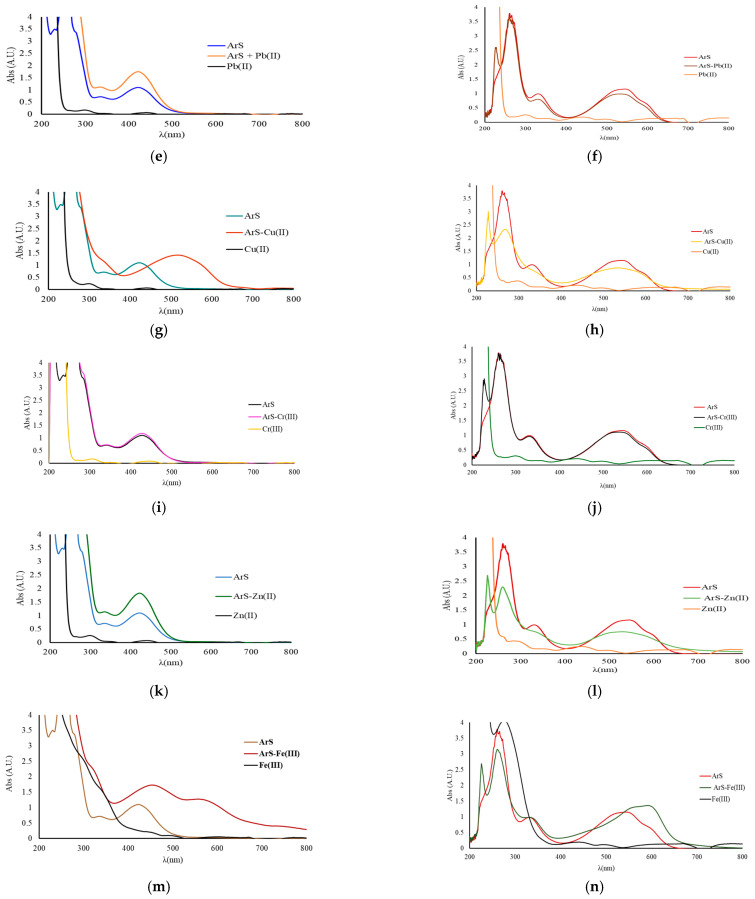
(**a**–**m**). Image of the liquid solutions used in the spectral analysis (**a**,**b**) and UV-Vis absorption spectra overlapped for ArS, M^n+^, and for the ArS-M^n+^ mixture (**c**–**m**). The ArS solution was obtained from 0.75 mL ArS 500 mg/L and 0.75 mL buffer solution of pH 4 in a 10 mL volumetric flask that was brought to the mark with ultrapure water. The ArS-M^n+^ in mixed solution was obtained from 0.5 mL M^n+^ (100 mg/L) together with 0.75 mL ArS (500 mg/L) and 0.75 mL acetate buffer pH = 4 in a 10 mL volumetric flask brought to the mark with ultrapure water (**c**,**e**,**g**,**i**,**k**,**m**). For testing complex formation in carbonate buffer solution at pH = 10, the same experimental procedure was applied (**d**,**f**,**h**,**j**,**l**,**n**).

To evaluate the complex formation between ArS and M^n+^, two pH levels were examined: pH 4 and 10. The interaction of ArS with M^n+^ was monitored spectrometrically across the 200–800 nm wavelength range. The results of ArS and the mixture of ArS-M^n+^ are presented in Figure 1a,b for pH 4 and 10.

Following UV-Vis studies for the mixed solutions of ArS-M^n+^, a new maximum of intensity is observed at pH = 4 and 10. The most significant interaction regarding complex formation is observed by a shift to lower wavelengths and lower intensity of the ArS-M^n+^ mixture spectra with respect to the ArS spectrum, suggesting the formation of a complex together with a color change.

At the same time, if we analyze each mixed solution presented in Figure 1a, at pH = 4, the above-mentioned behavior is only observed in the case of Cu^2+^ and Fe^3+^, both from spectra analysis (Figure 1g,m) and visual analysis of color (Figure 1a).

At pH = 10, the above-mentioned behavior is observed for all ArS-M^n+^ mixture spectra (Figure 1d,f,h,j,l,n), and also from the colors of solutions obtained, both suggesting the formation of complexes for the mixtures tested (Figure 1b).

### 3.2. Functionalization of Shredded Maize Stalk with ArS in Function of pH Solution

**Figure 2:**
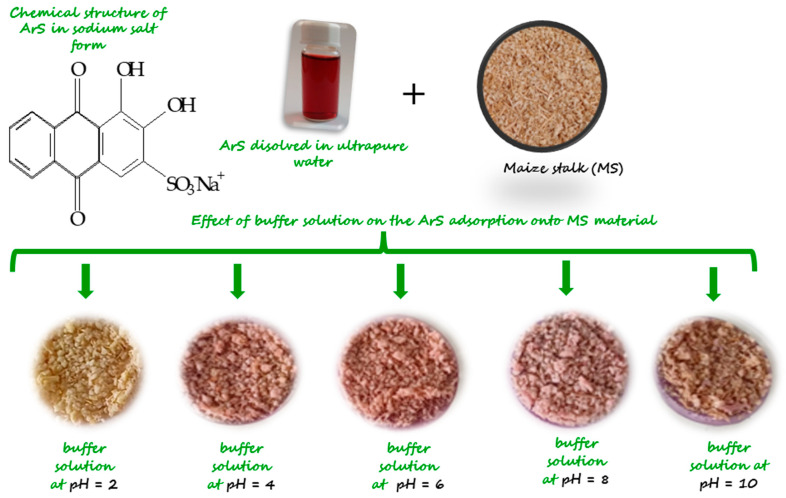
Image of MS-ArS obtained at pH = 2.0; 4.0; 6.0; 8.0 and 10.

**Figure 3:**
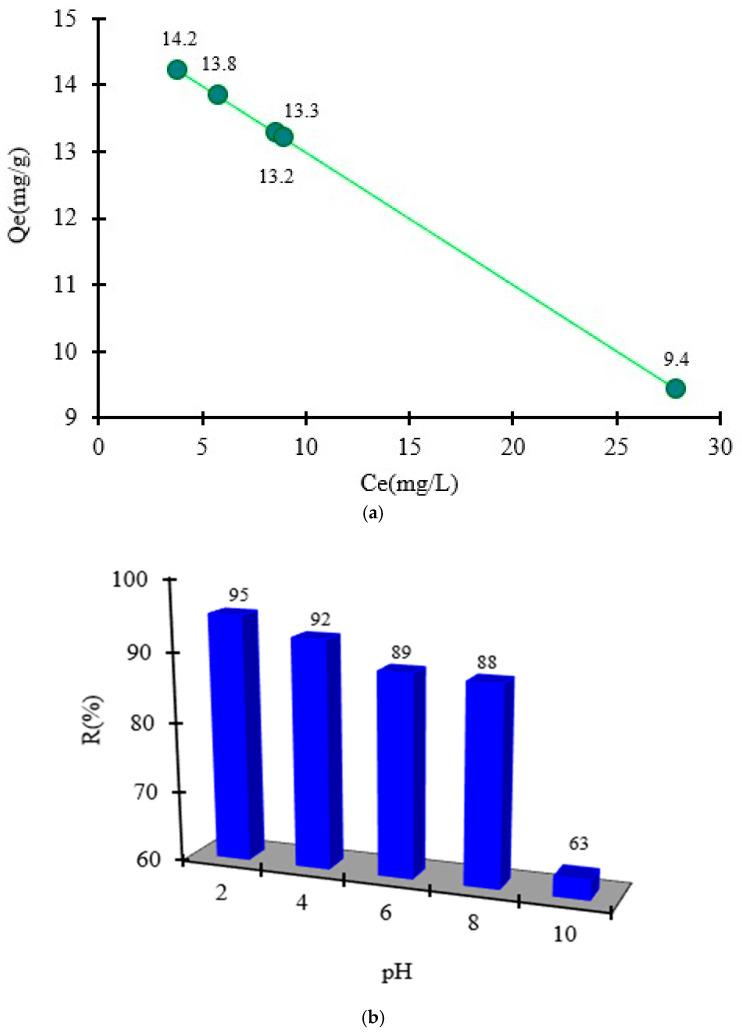
Quantity of ArS adsorbed onto MS mass in function of pH medium (**a**) and as a function of R(%) (**b**).

Because the organic compound ArS changes its structure when the pH changes, it was studied to see how much the pH change affects the retention of the functionalizing agent when it is adsorbed onto MS mass. Functionalization of MS with ArS using different solutions with pH = 2, 4, 6, 8, and 10 was studied, and the resulting solid phases are presented in Figure 2. During experimental studies the following adsorption capacities in function of pH medium was obtained for adsorption of ArS onto MS mass (Figure 3a) and were at pH = 2 (Q_e_ = 14.2 mg/g) > pH = 4 (Q_e_ = 13.8 mg/g) > pH = 6 (Q_e_ = 13.3 mg/g) > pH = 8 (Q_e_ = 13.2 mg/g) > pH = 10 (Q_e_ = 9.4 mg/g). Hence, the percentage removal of ArS gradually decreased from 95 to 63% with increasing the pH from 2 to 10. Analyzing the obtained results, it was observed that maximum adsorption of ArS on MS was obtained at pH = 2 (Q_e_ = 14.2 mg/g), and this pH value was used to obtain MS-ArS material for metal ions adsorption.

Taking into consideration the results obtained, the complexing mechanism can be explained as: ArS, an anionic compound having a sulfonic group, which is totally dissociated even at pH = 2. This behavior can be attributed to the fact that, in a strongly acidic environment, the hydroxyl groups present in the cellulose structure are protonated and engage in electrostatic interactions with the sulfonic group (SO_3_^−^) of the complexing agent ArS.

As the pH of the buffer solutions increases, deprotonation of the hydroxyl groups takes place, leading to a decrease in the degree of adsorption observed due to the electrostatic repulsions that occur when considering the behavior of ionizable groups at pH 4 and 6.

At a pH greater than 7, the adsorption process can be explained by considering the mass of the adsorbent. Aside from the specific adsorption processes associated with the ionizable groups present in the structure of the tested materials—particularly the hydroxyl groups—the overall charge of the adsorbent was close to zero. Therefore, the adsorption of ArS occurs through physical interactions or diffusion within the structure of the MS material. This may explain why the adsorption capacity of ArS on the MS mass was higher at pH 2 compared to the next pH values studied.

### 3.3. Proposed Mechanism for Adsorption of Metal Ions onto MS-ArS Mass

The functional groups present in the structure of the complexing agent can exhibit an affinity for metal ions by binding metal ions through a complexation mechanism [38,39].

**Figure 4:**
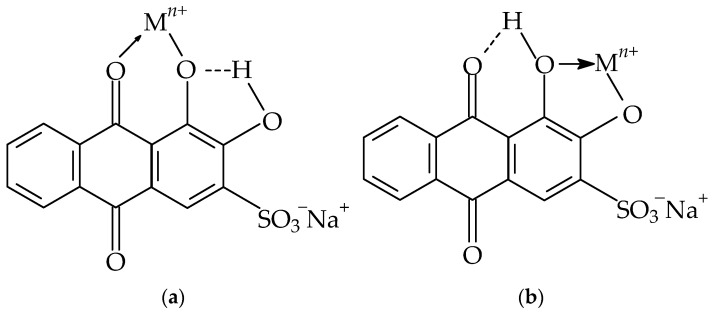
Chemical structure of ArS (**a**,**b**) and its forms in which it can complex the metals (Mn^2+^, Pb^2+^, Cu^2+^, Cr^3+^, Zn^2+^, Fe^3+^).

Thus, the adsorption mechanism between the metal and the complexing material can be explained by considering the following hypotheses: (i) the carbonyl (C=O) and hydroxyl (OH) groups present in the ArS structure may be responsible for the complexation of the Mn^2+^, Pb^2+^, Cu^2+^, Cr^3+^, Zn^2+^ and Fe^3+^ (Figure 4a,b); (ii) by adsorption through hydrogen bonding or Van der Waals forces and also, by diffusion into the porous structure of the complexing material, taking into consideration the ionic radius and metal electronegativity; (iii) by ion exchange mechanism between the −SO_3_^−^ group of ArS, which is not involved in the first adsorption step with MS; (iv) also, MS has in its structure three main biopolymers like: cellulose rich in OH and glycosidic C–O–C groups, second hemicellulose that have OH and carbonyl group (C=O) and lignin with phenolic –OH, aromatic rings, methoxy groups and carbonyl groups (C=O). Those groups can coordinate metal ions through the oxygen atom after deprotonation.

### 3.4. The pH Influence for the Metal Ions Adsorption onto MS-ArS

Literature data show that most materials retain pollutants at specific established pH values [16]. This is because the pH of the solution influences both the solubility of metal ions and the degree of dissociation of ionic/ionizable groups in the material structure. Thus, the functionalizing agent (ArS) contains OH and C=O groups in its structure, which change their degree of dissociation in function of the pH medium. Thus, for pH > 9, OH groups on the ArS are dissociated and can bind the metal ions present in the solution.

**Figure 5:**
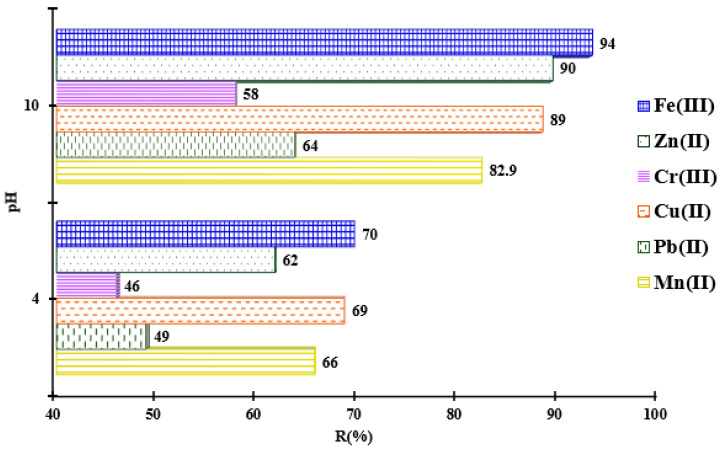
Adsorption of Mn^2+^, Pb^2+^, Cu^2+^, Cr^3+^, Zn^2+^, and Fe^3+^ in function of pH solution onto MS-ArS mass.

In order to avoid metal precipitation at high pH values, buffer solutions were used. Herein, the metal adsorption value increased from 66 to 83% for Mn^2+^, from 49 to 64% for Pb^2+^, from 69 to 89% for Cu^2+^, from 46 to 58% for Cr^3+^, from 62 to 90% for Zn^2+^ and from 70 to 94 for Fe^3+^ when the pH of the solution increased from 4 to 10, using MS-ArS (Figure 5). As one can observe, at higher pH, there was an increase in the removal rate of metals from the solution by complexation, and this can only take place at a certain pH value experimentally established. So, the pH of the aqueous solution influences the dissociation of functional groups in the structure of the adsorbent material as well as the speciation of metals in the aqueous solution. Thus, at pH < 4, the retention of metal ions was poorly favored due to the competitive sorption of H^+^ ions on the functionalized material, whose groups were protonated and unavailable for retaining metal ions from the solution. At the same time, as the pH of the solution increases, it intensifies the dissociation of OH groups, so that the surface of the material becomes more negative and adsorption is favored by electrostatic interactions between metal ions and the mass of the tested material. This was proven by the fact that the adsorption capacities determined at pH 10 were significantly higher than those determined at pH = 4 for all studied metal ions. Taking into account the results obtained at pH influence studies, the next experimental metal ions adsorption studies were focused only for pH = 10.

### 3.5. Kinetics Studies for Mixed Metal Ions

**Figure 6:**
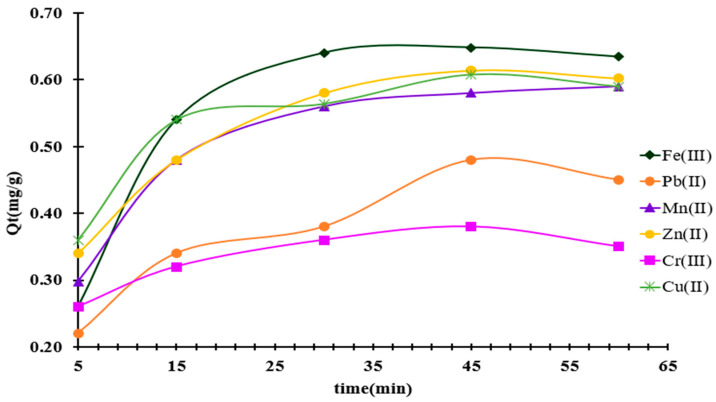
Effect of contact time on the removal of metal ions onto MS-ArS mass.

The contact time between the metal ion solution and MS-ArS required to reach equilibrium for best adsorption was also experimentally tested. The experimental results obtained when studying the influence of the contact time between Mn^2+^, Pb^2+^, Cu^2+^, Cr^3+^, Zn^2+^, and Fe^3+^ and MS-ArS in the range of 5–60 min are presented in Figure 6.

From Figure 6, it is observed that the retained metal amount per mass of MS-ArS increases with the increase in contact time between the two phases (liquid-solid). Thus, a more pronounced increase is observed during the initial stage, which ranged from 5 to 30 min. After this stage, the adsorption of metals becomes slower, and the Q_t_ values increase with 7% for Pb^2+^, 3% for Mn^2+^, 2% for Zn^2+^, and 3% for Cu^2+^, while for Fe^3+^ and Cr^3+^, the equilibrium is reached in the first 30 min. Based on these experimental observations, it was concluded that a contact time of 60 min is sufficient for retaining Pb^2+^, Mn^2+^, Zn^2+^, and Cu^2+^ on MS-ArS, whereas for Fe^3+^ and Cr^3+^, a contact time of only 30 min was necessary.

Based on the information provided, it was determined that a contact time of 60 min is appropriate to ensure that the system reaches equilibrium for all the metal ions studied in mixed solutions. This time allows sufficient interaction between the ions and the solution, leading to consistent and reliable experimental results when analyzing the behavior and adsorption or reaction characteristics of the metals involved.

The kinetic data were further fitted according to the Pseudo-first order model (Lagergren model) (Equation (4)), Morris Weber (Equation (5)), and Second order kinetic models type 1–4 (Equations (6)–(9)) [37,40,41]. The results of the constants calculated for each model are presented in Table 2.

(4) $$\log\left(Q_{e}-Q_{t}\right)=\log Q_{e}-\left(\frac{k}{2303}\right)t$$

(5) $$Q_{t}=k_{id}{\left(t\right)}^{0.5}\;+C$$

(6) $$\frac{t}{Q_{t}}=\frac{1}{k_{2\;}(Q_{e}\times Q_{e})\;}+\frac{t}{Q_{e}}\;(\mathrm{Type}\;1)$$

(7) $$\;\;\;\;\frac{1}{Q_{t}}=\frac{1}{Q_{e}}+\frac{1}{k_{2\;}\;(Q_{e}\times Q_{e})}\;\frac{1}{t}\;(\mathrm{Type}\;2)$$

(8) $$Q_{t}=Q_{e}-\frac{1}{k_{2\;}\;Q_{e\;\;}\;}(\frac{Q_{t}}{t})\;(\mathrm{Type}\;3)$$

(9) $$\frac{Q_{t}}{t}=k_{2\;}(Q_{e}\times Q_{e})-k_{2}(Q_{e}\times Q_{t})\;(\mathrm{Type}\;4)$$

where *k* (min^−1^) is the rate constant of the adsorption process, *Q_e_* (mg/g) represents the equilibrium binding capacity of MS-ArS; *C* (mg/L) constant determined from the intercept and represents the concentration of M^n+^ at the surface of the complexing material; *K_id_* (min^−1^) value of the intraparticle diffusion constant; *k_2_* (g/(mg min)) is the rate constant of the second-order kinetic model.

The agreement between the experimental data and the applied model was evaluated based on the correlation coefficients (R^2^). As can be seen from Table 2, the results of the R^2^ for the linear representations of the studied kinetic models were higher for the second-order kinetic model. This model predicts the adsorption capacity at equilibrium, *Q_e_ calc.*, when compared to the experimentally obtained data, *Q_e_ exp*. The results suggest that the adsorption mechanism is given by the second-order kinetic model and the adsorption rate is controlled by chemisorption. However, there were higher values of *Q_e_ calc*., compared to the *Q_e_ exp* experimentally determined.

### 3.6. Ishoterm Studies for Mixed M^n+^

**Figure 7:**
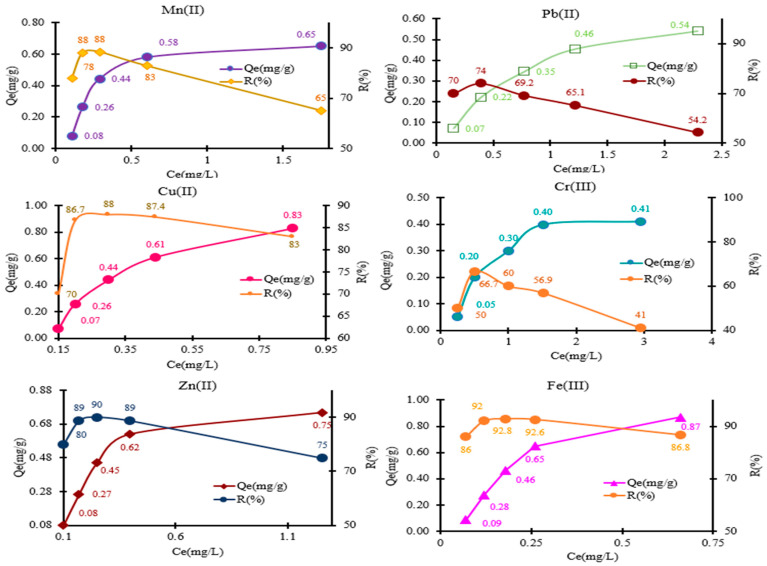
Adsorption of Mn^2+^, Pb^2+^, Cu^2+^, Cr^3+^, Zn^2+^, and Fe^3+^ in the MS-ArS mass at pH = 10 of the tested solution.

To represent the distribution profile of metal ions between the liquid and solid phases, adsorption isotherms were experimentally plotted. For this purpose, the *Q_e_* values (mg/g) were graphically represented as a function of the concentration remaining in the supernatant solution at equilibrium (*C_e_*, mg/L) [40,41,42,43,44]. Analyzing the isotherms presented in Figure 7, it can be observed that the adsorption of metal ions is nonlinear over the whole range of concentrations tested, which was varied from 0.5 to 5 mg/L in the mixture solution for all metal ions studied.

The testing of the complexing material was conducted using the batch method, following the experimental conditions presented in Section 2.8. The adsorption behavior for each metal studied in mixed solutions was as follows: for Mn^2+^ ions, the amounts retained were 0.08, 0.26, 0.44, 0.58, and 0.65 mg/g. For Pb^2+^, the obtained adsorption values were 0.07, 0.22, 0.35, 0.46, and 0.54 mg/g. In the case of Cu^2+^, the amounts recorded were 0.07, 0.26, 0.26, 0.44, 0.61, and 0.83 mg/g. For Cr^3+^ ions, the calculated adsorption capacities ranged from 0.05 to 0.41 mg/g, with values of 0.05, 0.20, 0.30, 0.40, and 0.41 mg/g, respectively. Additionally, during the investigation of Zn^2+^ retention on MS-ArS, the following *Q_e_* values (mg/g) were obtained: 0.08, 0.26, 0.45, 0.62, and 0.75 mg/g. The amounts (*Q_e_*, mg/g) of metal ions retained on 0.05 g of MS-ArS were 0.09, 0.28, 0.46, 0.65, and 0.87 mg/g for Fe^3+^.

The adsorption capacities of the tested metal ions varied, as shown in Figure 7. The highest adsorption capacity was observed for Fe^3+^ at 0.87 mg/g, while the lowest was for Cr^3+^ at 0.44 mg/g. Taking into account the results presented, in a mixed solution, the order of adsorption capacity for the metals was as follows: Fe^3+^ (0.87 mg/g) > Cu^2+^ (0.83 mg/g) > Zn^2+^ (0.75 mg/g) > Mn^2+^ (0.65 mg/g) > Pb^2+^ (0.54 mg/g) > Cr^3+^ (0.41 mg/g). The observed variations in adsorption capacities for the six metals studied can be attributed to differences in their ionic radius and electronegativities (Table 1).

These physicochemical properties influence effectively each interaction of metal ions with the MS-ArS material effectively. Specifically, a smaller ionic radius can facilitate closer interaction and stronger binding with the adsorbent’s active sites, while higher electronegativity may enhance the affinity between the metal ions and the surface of functional groups. Consequently, these factors together determine the efficiency of metal ion adsorption onto MS-ArS mass.

The experimental results indicate that as the concentration of M^n+^ increases within the studied range (0.5–5 mg M^n+^/L), there is a corresponding decrease in the metal retained percentage (R%). This trend is attributed to the increasing number of M^n+^ ions competing for a limited number of functional groups available in the structure of the complexing material. So, when M^n+^ concentration rises, the finite binding sites become saturated or less effective in retaining additional M^n+^, leading to a reduction in overall retention efficiency, as illustrated in Figure 7.

In the Supplementary Material, the literature studies on the use of functionalized cellulose materials are presented. From the analysis of the adsorption data, it is found that MS-ArS has a low adsorption capacity compared to other materials presented in Table S1. It should also be mentioned that the data from Table S1 are obtained under different experimental conditions, especially regarding the initial concentration tested, which ranged from 700 to 1800 mg/L. Considering that the literature studies test high concentrations of metals for adsorption, testing the medium or moderate concentrations found in most wastewaters can be addressed with the material (MS-ArS) developed in this study.

Data obtained at equilibrium was fitted in the next step, taking into consideration Langmuir (Equations (10) and (11)), Freundlich (Equation (12)), and Dubinin–Radushkevich (Equations (13)–(15)) models [40,41].

(10) $$\frac{C_{e}}{Q_{e}}=\frac{1}{bQ_{0}}+\frac{C_{e}}{Q_{0}}$$

(11) $$R_{L}=\frac{1}{1+bC_{0}}$$

(12) $$\ln Q_{e}=\ln K_{f}+\frac{1}{n}{\mathrm{lnC}}_{e}$$

(13) $$\ln Q_{e}=\ln q_{m}-\beta \varepsilon^{2}\;$$

(14) $$\varepsilon =\mathrm{RTln}(1+\frac{1}{C_{e}})$$

(15) $$the\;E=\frac{1}{\sqrt{2\beta }}$$

where *Q*_0_—maximum adsorption of MS-ArS, *b*—Langmuir constant correlated with the adsorption capacity of MS-ArS, *C*_0_ (mg/L) is the highest initial concentration of M^n+^ adsorbed by the MS-ArS at equilibrium; *R_L_*: separation factor; the value of the separation factor *R_L_*, shows whether the adsorption process is favorable 0 < *R_L_* < 1, unfavorable *R_L_* > 1, linear *R_L_* = 1 and irreversible *R_L_* = 0; *K_f_*—Freundlich constant, *n*—constant correlated with the energetic heterogeneity of the adsorption sites. *q_m_* is the adsorption capacity of a theoretical monolayer (mg/g); *β* is the constant of adsorption energy (mol^2^/J^2^), *Ɛ* is the Polanyi potential, and *E* (KJ/mol) is the mean of the free energy.

To describe the most appropriate isotherm, linear regression was applied. The applicability of the isotherm equation was analyzed based on the correlation coefficients. The constants calculated based on the Langmuir, Freundlich, and Dubinin–Radushkevich equations are presented in Table 3. The correlation coefficients for the graphical representation of *C_e_*/*Q_e_* as a function of C_e_ for Pb^2+^ and Mn^2+^ were 0.9405 and 0.8570, results suggesting that the adsorption data of these ions are in concordance with the Langmuir isotherm. For the Freundlich isotherm, the graphical representation of log *Q_e_* vs. *C_e_* gives values of correlation coefficients equal to 0.8490 for Fe^3+^, 0.7468 for Zn^2+^, 0.8230 for Cr^3+,^ and 0.7993 for Cu^2+^. These values indicate that their adsorption was consistent with the Freundlich isotherm theory. Also, the *E* (KJ/mol) values > 16 KJ/mol determined applying the Dubinin–Raduschevich model showed that M^n+^ is retained by a chemical adsorption on the MS-ArS (Table 3).

### 3.7. Studies on the Regeneration of Complexing Material Loaded with Mn^2+^, Pb^2+^, Cu^2+^, Cr^3+^, Zn^2+^ and Fe^3+^

One of the key opportunities for improvement in the adsorption process lies in enhancing the methods for regenerating and recovering saturated adsorbents. By focusing on these aspects, we can increase efficiency and sustainability in the overall process [45,46,47].

**Figure 8:**
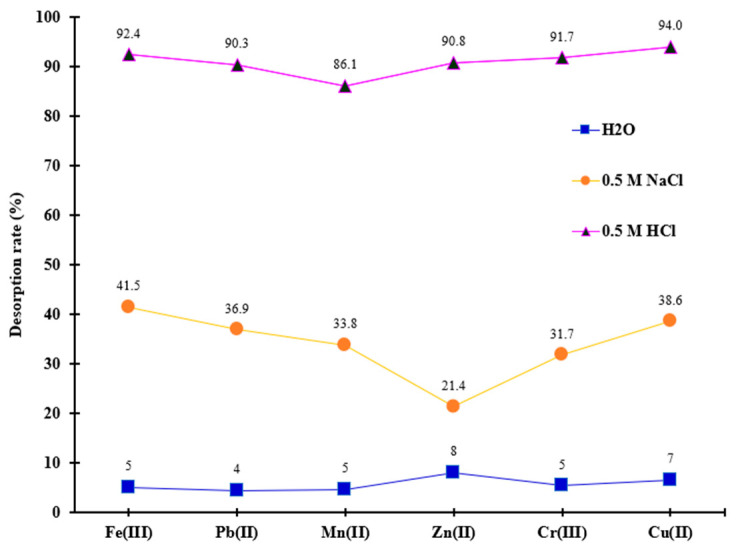
Desorption capacities for metal ions removal from ArS-MS mass after adsorption studies.

In this study, metal recovery from saturated material was achieved through chemical means using 0.5 M NaCl and HCl, as well as through heat treatment in hot water maintained at 45 °C throughout the desorption experiment. It was observed, as it is shown in Figure 8, that the desorption rate of retained metals was lower when using hot water for regeneration.

In contrast, metal desorption began to increase significantly with the application of the 0.5 M NaCl and HCl solutions. The desorption percentages obtained with 0.5 M NaCl were below 50% (Figure 8).

Notably, the 0.5 M HCl solution exhibited the most pronounced desorption effect, yielding percentages for Mn^2+^, Pb^2+^, Cu^2+^, Cr^3+^, Zn^2+^, and Fe^3+^ that reached up to 94%. These results indicate that the chemical adsorption resulting from the complex reaction between metals and functional groups of MS-ArS is the predominant mechanism at work, which explains the low desorption efficiency with hot water.

The regeneration mechanisms for NaCl and HCl were fundamentally similar. In both cases, Na^+^ and H^+^ ions engage to replace the metal ions that were adsorbed on the surface of the complexing material. However, the HCl solution demonstrated a greater desorption capacity compared to the NaCl solution. Among the three desorption agents tested, 0.5 M HCl was identified as the most effective reagent for regenerating the MS-ArS exhausted with metal ions, suggesting that the complexing material shows a higher affinity for H^+^ ions compared to Na^+^ ions.

The solutions obtained after the regeneration study were also tested spectrometrically to detect the concentration of ArS that could be released into solution by desorption agents. Following the analysis of the supernatant solutions, it was found that the ArS remains fixed in the MS mass because the percentages determined spectrometrically of ArS were detected below the detection limit of the UV-Vis method. These results suggested that the MS-ArS material can be used in multiple adsorption–desorption cycles without losing its complexing properties.

### 3.8. Reusability of Complexing Material

**Figure 9:**
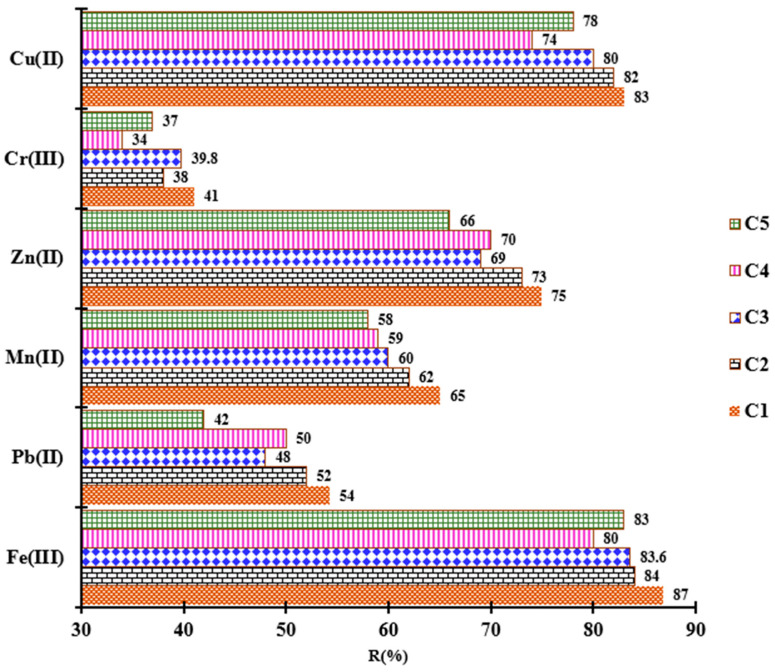
Reusability of complexing material for M^n+^ adsorption in five adsorption studies.

The concept of the Circular Economy emphasizes the importance of keeping resources and materials in use for as long as possible, transforming waste into valuable products for further use. For regeneration of the complexing material, a 0.5 M HCl solution was chosen, considering the subsequent experiment. The percentages removed for each adsorption cycle studied (C1–C5) are presented in Figure 9. It is observed that the retained percentages begin to decrease slightly with the increase in regeneration cycles, and the removed percentages have changed (with a variation of up to 12%). This aspect can be attributed to the oxidizing and corrosive properties of HCl. Thus, after several desorptions, the functional groups are slightly oxidized, and the porous structure of the complexing material is reduced, leading to a decrease in the adsorption centers [48,49]. All these results demonstrate that the complexing material has considerable performance after several adsorption/desorption cycles, substantially reducing the production cost of the developed material and the cost of treating wastewater polluted with metals.

### 3.9. FTIR-ATR Studies

The FTIR spectra for the maize stalk (MS), maize stalk loaded with ArS (MS-ArS), and the metal-loaded complex (MS-ArS-M^n+^) are illustrated in Figure S1a,b of the Supplementary Material.

The efficiency of the adsorption capacity of the MS-ArS system, given by the adsorption isotherms (in monolayer, on heterogeneous surface with different binding energies), is influenced by modifications of the adsorption mechanism induced by ArS loading.

The peak shift in the wavelength range 3300–1000 cm^−1^ is direct spectral evidence of the chemical interaction between the maize stalk and the adsorbate, signaling some specific processes [50].

The spectrum of MS exhibits prominent absorption bands at 3338 cm^−1^ and 2902 cm^−1^, corresponding to the stretching vibrations of OH and CH groups, respectively.

These shifts in the OH group towards lower values correspond to the formation of strong hydrogen bonds and even a complexation between the hydroxyl groups of cellulose/lignin and ArS molecules. In the region of the CH groups, changes occur in the aliphatic structure of the biomass due to the rearrangement of natural polymer chains to accommodate the adsorbate. Induced chemical phenomena, such as charge transfer, indicate an elongation of the O-H or C=O bond, which confirms the stable retention of the pollutant, while ion exchange occurs as a result of the replacement of the proton (H^+^) with an ion from the ArS solution.

These are characteristic of the macro-components—cellulose, hemicellulose, and lignin—inherent to the maize stalk structure. The presence of abundant surface OH groups suggests a high capacity for protonation in acidic environments, facilitating the electrostatic binding of negatively charged ArS molecules. Additionally, the peak at 1513.95 cm^−1^ is attributed to the skeletal vibrations of the aromatic lignin rings.

The decrease in intensity with the shift in the position of the bands shows that the adsorption process chemically modifies the surface of the maize stalk, so that the OH groups no longer vibrate freely, being involved in new bonds. A shielding effect (weaker signal) appears as a result of the presence/deposition of the ArS layer on the surface of the biopolymers, masking the vibrations of the cellulose chain, but also a change in polarity around these groups.

In conclusion, the simultaneous action in the range of 3300–1000 cm^−1^ proves a chemical reaction on the surface of the groups that have undergone structural changes and not just a physisorption. The decrease in the intensity of the C=O band (1718 cm^−1^) coupled with its shift suggests that oxygen has formed a complex with the adsorbate, redistributing the electrical charges within the carboxyl group.

A comprehensive summary of these functional groups is provided in Table 4.

Upon the adsorption of ArS onto the MS biomass, a significant decrease in band intensity was observed across the spectrum. Specifically, the intensities of the ν(OH), ν(C=C), and ν(C=O) bands were attenuated. This overall reduction in spectral intensity confirms a strong interfacial interaction between the ArS molecules and the maize stalk matrix.

Following the interaction of the MS-ArS complex with metal ions in a mixed solution, distinct spectral shifts were observed, indicating the specific functional groups involved in complexation. The ν(OH) band shifted from 3342 cm^−1^ (MS-ArS) to 3336 cm^−1^ after metal uptake. This shift toward lower wavenumbers indicates that the oxygen atoms within the hydroxyl groups participate directly in metal ion binding. The stretching frequency of the carbonyl group ν(C=O), from the ArS structure, shifted from 1601 cm^−1^ to 1589 cm^−1^. This transition confirms that the carbonyl oxygen atoms are involved in the coordination of the metal ions. Changes were also noted in the ν(C-O) region, where the peak shifted from 1036 cm^−1^ to 1033 cm^−1^. This variation reflects the interaction between the metal ions and the oxygen atoms of the polysaccharide units.

## 4. Conclusions

During this study, we successfully synthesized a novel material by functionalizing maize stalk (MS) with Alizarine Red (ArS). The process consisted of mixing MS with ArS, an organic reagent characterized by its complexing groups, in a discontinuous manner at pH 2.

Also, complex formation between ArS and M^n+^ at pH 4 and 10 was tested, resulting in significant structural and color changes that confirmed the formation of complex compounds initially in liquid solution.

The results show that the adsorption capacity of MS-ArS increases in correlation with the M^n+^ concentration gradient, and the time required to reach equilibrium was relatively short. The adsorption process of M^n+^ onto MS-ArS followed the Pseudo-Second order model type 1. The mechanism of M^n+^ adsorption onto MS-ArS mass with the Langmuir and the Freundlich models was in agreement.

M^n+^ desorption and regeneration of the complexing material for reuse were performed with 0.5 M HCl. After five adsorption/desorption studies, MS-ArS maintains good metal ion adsorption and exhibits high cost-effectiveness.

These results demonstrated the efficacy of functionalization in improving the performance of cellulose material, leading to further applications and improvements in the performance of cellulose material.

## Figures and Tables

**Table 1:** Characteristics of M^n+^ studied.

Metal	Mn^2+^	Pb^2+^	Cu^2+^	Cr^3+^	Zn^2+^	Fe^3+^
Electron Configuration	[Ar] 3d^5^ 4s^2^	[Xe] 4f^14^ 5d^10^ 6s^2^ 6p^2^	[Ar] 3d^10^ 4s^1^	[Ar] 3d^5^ 4s^1^	[Ar] 3d^10^ 4s^2^	[Ar] 3d^6^ 4s^2^
Electronegativity	1.55	2.33	1.90	1.66	1.65	1.83
Oxidation Number	+2	+2	+2	+3	+2	+3
Atomic radius (Å)	1.79	1.9	1.57	1.85	1.53	1.72
Ionic radius (Å)	0.67	1.19	0.73	0.62	0.74	0.55

**Table 2:** Kinetic constants of M^n+^ adsorption on the MS-ArS mass.

Kinetic Models	Fe^3+^	Pb^2+^	Mn^2+^	Zn^2+^	Cr^3+^	Cu^2+^
Pseudo-first order model (Lagergren model)						
*k*_1_ (min^−1^)	0.09	0.05	0.08	0.08	0.03	0.06
*Q_e_ calc.* (mg/g)	2.78	3.73	2.43	2.72	16.4	5.09
*Q_e_ exp.* (mg/g)	0.63	0.45	0.59	0.60	0.35	0.59
R^2^	0.7290	0.9785	0.9965	0.9654	0.3401	0.8492
Morris Weber model						
*C*	0.21	0.14	0.24	0.27	0.24	0.33
*K_id_*	0.07	0.04	0.05	0.05	0.02	0.04
R^2^	0.7525	0.9032	0.8558	0.8836	0.7467	0.7691
Pseudo-second order (Type 1)						
*k*_2_ (g/(mg∙min))	0.22	0.25	0.29	0.32	1.60	0.51
*Q_e_ calc* (mg/g)	0.72	0.52	0.65	0.66	0.37	0.63
R^2^	0.9901	0.9850	0.9995	0.9979	0.9937	0.9979
Pseudo-second order (Type 2)						
*k*_2_ (g/(mg∙min))	0.11	0.32	0.25	0.32	1.13	0.39
*Q_e_ calc* (mg/g)	0.83	0.50	0.66	0.65	0.38	0.65
R^2^	0.9795	0.9772	0.9982	0.9904	0.9520	0.9843
Pseudo-second order (Type 3)						
*k*_2_ (g/(mg∙min))	758	2179	1000	919	5885	1100
*Q_e_ calc* (mg/g)	0.35	0.22	0.33	0.36	0.28	0.41
R^2^	0.5119	0.7735	0.7128	0.7476	0.5484	0.6058
Pseudo-second order (Type 4)						
*k*_2_ (g/(mg∙min))	0.12	0.27	0.25	0.99	1.00	0.38
*Q_e_ calc* (mg/g)	0.82	0.52	0.66	0.39	0.38	0.65
R^2^	0.8633	0.9035	0.9928	0.9741	0.9054	0.9564

**Table 3:** Constants of Langmuir, Freundlich, and Dubinin–Radushkevich adsorption isotherms regarding M^n+^ adsorption on ArS-MS.

Isotherm Models	Fe^3+^	Pb^2+^	Mn^2+^	Zn^2+^	Cr^3+^	Cu^2+^
Langmuir						
*Qo* (mg/g)	2.74	0.88	0.90	1.35	0.81	1.55
*b* (L/mg)	0.77	0.74	1.76	1.18	0.44	0.49
R^2^	0.2285	0.9405	0.8570	0.5094	0.5302	0.1000
*R_L_*	0.23	0.33	0.15	0.18	0.53	0.32
Freundlich						
*K_F_*	1.54	2.88	1.75	1.09	4.17	1.42
1/*n*	0.90	0.71	0.60	0.81	0.83	1.28
*n*	1.11	1.40	1.66	1.24	1.21	0.78
R^2^	0.8490	0.9343	0.7257	0.7468	0.8230	0.7993
Dubinin–Radushkevich						
*q_m_* (mg/g)	1.32	1.84	1.2	1.10	2.10	1.52
*β* (mol^2^/kJ^2^)	1.2 × 10^−7^	8 × 10^−8^	6 × 10^−8^	7 × 10^−8^	1 × 10^−7^	1 × 10^−7^
*E* (KJ/mol)	2887	2500	2887	2673	2236	2237
R^2^	0.9659	0.9913	0.9291	0.9482	0.9874	0.9166

**Table 4:** Functional groups of MS before and after functionalization, and subsequently loaded with metal ions.

Functional Groups	MS (cm^−1^)	MS-ArS (cm^−1^)	MS-ArS-M^n+^ (cm^−1^)
ν OH	3338.33	3343	3336
ν CH	2902.44	2901	2896
ν C=O	1718.10	1601	1589
ν C=C	1513.95	1508	1508.3
ν C-H	1369.29	1317	1317
ν C-O	1035.19	1036	1033

## Data Availability

The original contributions presented in this study are included in the article. Further inquiries can be directed to the corresponding author.

## Supplementary Materials

The following supporting information can be downloaded at: https://www.mdpi.com/article/10.3390/polym18060712/s1.
