# Supplementary material for: New Sustainable Material for Metal Ions Removal: Adsorption Mechanism and Technological Innovations
Source: Polymers (Basel). 2026 Mar 14;18(6):712. doi: 10.3390/polym18060712 (PMC13029952; doi:10.3390/polym18060712)
Supplement: Supplementary file 1 [file polymers-18-00712-s001.zip › polymers-4189628-supplementary.pdf]

polymers

MDPI

Supplementary Material

New Sustainable Material for Metal Ions Removal: Adsorption Mechanism and Technological Innovations

Luoana Florentina Pascu [1], Toma Galaon [1,2], Adriana Mariana Borș [3] and Nicoleta Mirela Marin [1,2,4,*]

[1] National Research and Development Institute for Industrial Ecology ECOIND, Street Podu Dambovitei No. 57-73, District 6, 060652 Bucharest, Romania; luoanapascu@yahoo.com (L.F.P.); tomagalaon@yahoo.com (T.G.)
[2] Department of Analytical and Physical Chemistry, University of Bucharest, 4-12 Regina Elisabeta Bd., 030018 Bucharest, Romania
[3] National Institute for R&D for Optoelectronics-Subsidiary, Research Institute for Hydraulics and Pneumatics—INOE 2000-IHP, 040558 Bucharest, Romania; bors.ihp@fluidas.ro
[4] Department of Oxide Materials Science and Engineering, National University of Science and Technology Politehnica Bucharest, 1–7 Gh. Polizu, 060042 Bucharest, Romania
* Correspondence: nicoleta.marin@incdecoind.ro

Table S1. Comparison of MS-ArS with different cellulosic adsorbents use for metal ions removal.

Supported material (adsorbent)	Modifying ligand	Metal ions Adsorption capacities (mg/g)						C_i (mg/L)	References
		Mn^{2+}	Pb^{2+}	Cu^{2+}	Cr^{2+}	Zn^{2+}	Fe^{2+}		
Pine wood	Poly(amidoxime) ligand	-	306	335	-	-	281	1500	[1]
Corn stalk cellulose	poly (amidoxime) ligand	-	-	310	205	-	280	1800	[2]
Corn stalk cellulose	-NH–/-NH_2 and C-S/C=S	-	241	152	-	-	-	500	[3]
Lignocellulose (fallen leaves)	Alkalized lignocellulose	-	-	-	-	20.04	-	1200	[4]
Lignocellulose (fallen leaves)	Xanthated lignocellulose	-	-	-	-	36.54	-	1200	[4]
Lignocellulose (fallen leaves)	Carboxylated lignocellulose	-	-	-	-	47.10	-	1200	[4]
Sugarcane bagasse	Poly(sodium acrylic acid) hydrogel (CE–PAANa)	-	-	106	333	-	-	700	[5]
Maize stalk	Alizarin red S	0.65	0.54	0.83	0.41	0.75	0.87	5	This study

FTIR-ATR Studies

The FTIR spectra of the maize stalk (MS), maize stalk loaded with ArS (MS-ArS), and the metal-loaded complex (MS-ArS-M^{n+}) are illustrated in Figure S1 a,b

 https://doi.org/10.3390/polym18060712

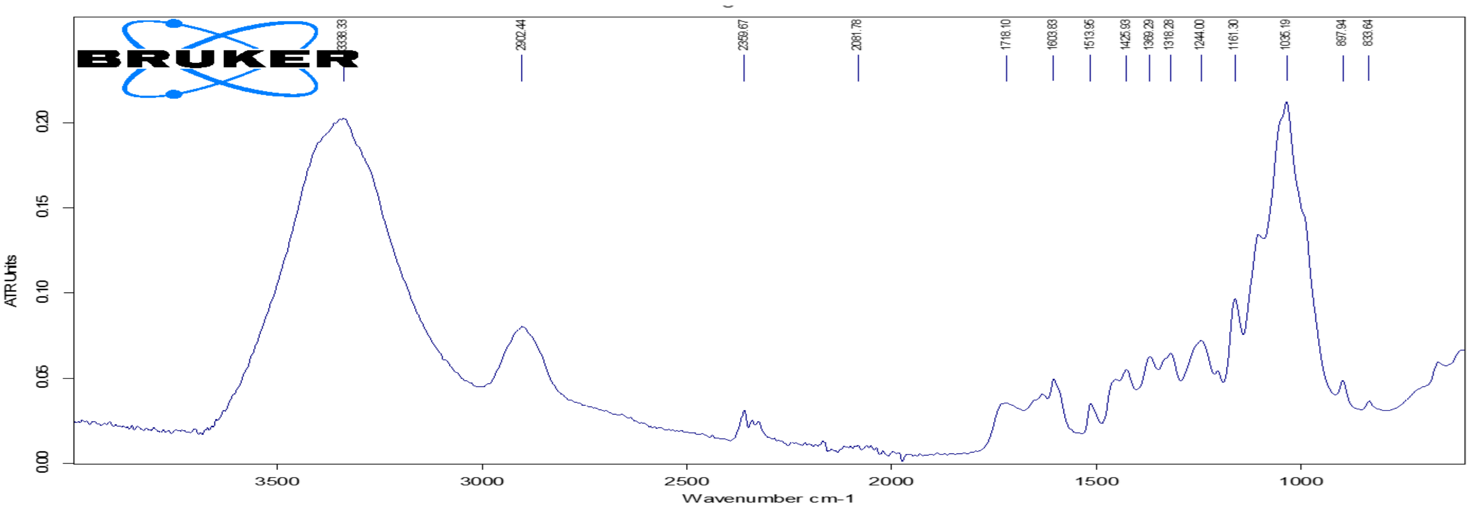

Figure S1a. FTIR spectrum of MS

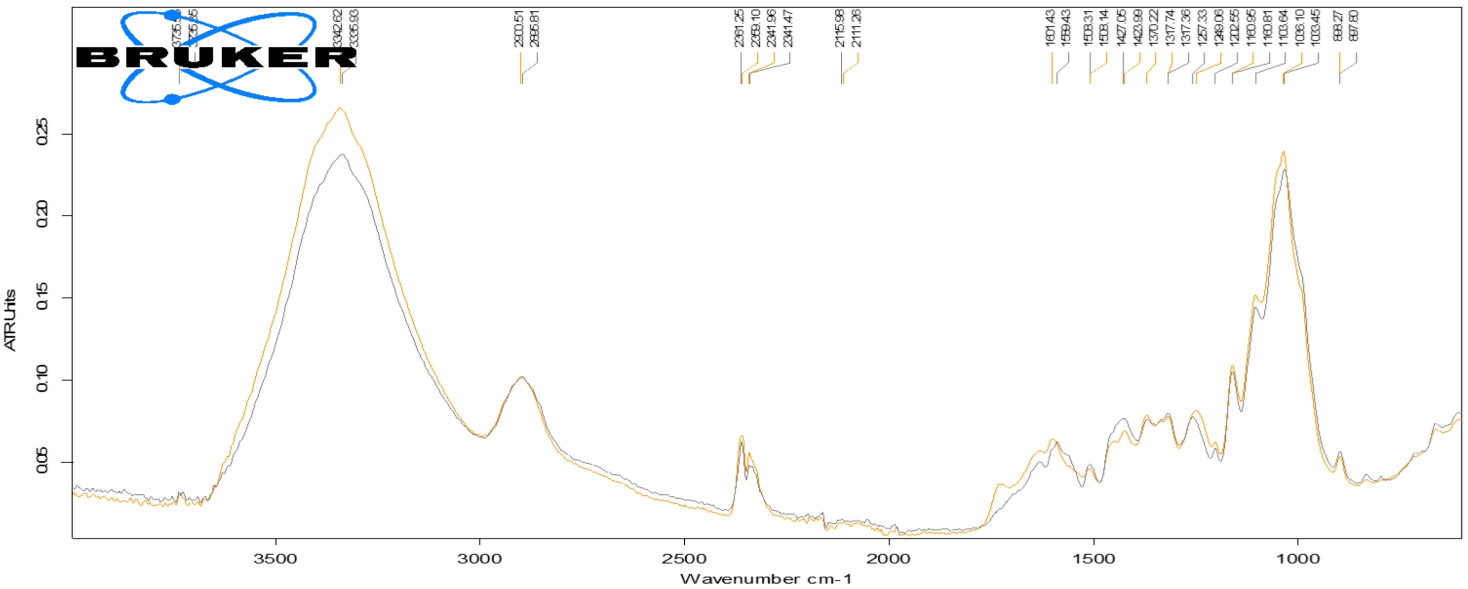

Figure S1b. FTIR spectrum of MS-ArS (orange spectra) MS-ArS loaded with metal ions in mixed solution (black spectra)

References

1. Rahman, M. L.; Aiman, M. A.; Sarjadi, M. S.; Arshad, S. E.; Sarkar, S. M.; Kumar, S., Poly(amidoxime) chelating ligand from pine wood cellulose for eco-friendly toxic metals extraction from water sources. *Results in Chemistry* **2025,** *15*, 102198.
2. Rahman, M. L.; Shamrih, S. A.; Azlyzan, N. A.; Sarjadi, M. S.; Arsad, S. E.; Sarkar, S. M.; Kumar, S., Removal of heavy metal ions from wastewater using modified cornstalk cellulose-derived poly (amidoxime) ligand. *Carbohydrate Polymer Technologies and Applications* **2025,** *9*, 100633.
3. Liu, Y.; Fan, H.; Wang, X.; Zhang, J.; Li, W.; Wang, R., Controllable synthesis of bifunctional corn stalk cellulose as a novel adsorbent for efficient removal of Cu2+ and Pb2+ from wastewater. *Carbohydrate Polymers* **2022,** *276*, 118763.
4. Dang, J.; Wang, H.; Wang, C., Adsorption of toxic zinc by functionalized lignocellulose derived from waste biomass: Kinetics, isotherms and thermodynamics. *Sustainability* **2021,** *13* (19), 10673.
5. Li, F.; Xie, Z.; Wen, J.; Tang, T.; Jiang, L.; Hu, G.; Li, M., Synthesis of cellulose–poly (acrylic acid) using sugarcane bagasse extracted cellulose fibres for the removal of heavy metal ions. *International Journal of Molecular Sciences* **2023,** *24* (10), 8922.
